# 17β-Estradiol Abrogates TNF-α-Induced Human Brain Vascular Pericyte Migration by Downregulating miR-638 via ER-β

**DOI:** 10.3390/ijms252111416

**Published:** 2024-10-24

**Authors:** Lisa Kurmann, Giovanna Azzarito, Brigitte Leeners, Marinella Rosselli, Raghvendra K. Dubey

**Affiliations:** 1 Department of Reproductive Endocrinology, University Hospital Zurich, 8952 Schlieren, Switzerland; kurmann_lisa@gmx.ch (L.K.); giovanna.azzarito@usz.ch (G.A.); brigitte.leeners@usz.ch (B.L.); marinella.rosselli@usz.ch (M.R.); 2 Department of Pharmacology & Chemical Biology, University of Pittsburgh, Pittsburgh, PA 15219, USA

**Keywords:** miRNAs, miR638, migration, pericytes, BBB, neurovascular unit, TNF-α, estrogen, estrogen receptor β, trauma, inflammatory cytokines

## Abstract

Pericytes (PCs) contribute to brain capillary/BBB integrity and PC migration is a hallmark for brain capillary leakage following pro-inflammatory insults. Estradiol promotes endothelial barrier integrity by inhibiting tumor necrosis factor-alpha (TNF-α)-induced PC migration. However, the underlying mechanisms remain unclear. Since micro-RNAs (miRs) regulate BBB integrity and increases in miR638 and TNF-α occur in pathological events associated with capillary leakage, we hypothesize that TNF-α mediates its capillary disruptive actions via miR638 and that estradiol blocks these actions. Using quantitative reverse transcription PCR, we first assessed the modulatory effects of TNF-α on miR638. The treatment of PCs with TNF-α significantly induced miR638. Moreover, transfection with miR638 mimic induced PC migration, whereas inhibitory miR638 (anti-miR) abrogated the pro-migratory actions of TNF-α, suggesting that TNF-α stimulates PC migration via miR638. At a molecular level, the pro-migratory effects of miR638 involved the phosphorylation of ERK1/2 but not Akt. Interestingly, estradiol downregulated the constitutive and TNF-α-stimulated expression of miR638 and inhibited the TNF-α-induced migration of PCs. In PCs treated with estrogen receptor (ER) ER-α, ER-β, and GPR30 agonists, a significant downregulation in miR638 expression was solely observed in response to DPN, an ER-β agonist. DPN inhibited the pro-migratory effects of TNF-α but not miR638. Additionally, the ectopic expression of miR638 prevented the inhibitory effects of DPN on TNF-α-induced PC migration, suggesting that interference in miR638 formation plays a key role in mediating the inhibitory actions of estradiol/DPN. In conclusion, these findings provide the first evidence that estradiol inhibits TNF-α-induced PC migration by specifically downregulating miR638 via ER-β and may protect the neurovascular unit during injury/stroke via this mechanism.

## 1. Introduction

Brain capillaries play a critical role in maintaining physiological brain function by acting as specialized and selective barrier between blood and the brain, termed blood–brain barrier (BBB) [1]. Brain capillary endothelial cells (ECs) were primarily believed to form the BBB. However, current evidence suggests the dynamic participation of multiple cell types and mechanisms within the neurovascular unit (NVU) in forming the BBB [2]. In this context, astrocytes, pericytes (PCs), neurons, glial cells and extracellular matrix components lining next to the capillary EC layer orchestrate the formation of the functional BBB [3,4,5,6]. Importantly, recent evidence from patho-physiologies associated with capillary leakage [7,8,9] has shed light on the critical role of PCs in maintaining BBB integrity and function. Moreover, the relevance of PC dysfunction with regard to various neurovascular disorders has been demonstrated in several studies [3,4], thereby highlighting the importance of PCs in promoting BBB capillary integrity.

PCs are vascular mural cells that share the same basement membrane with ECs. They are thought to stem from the same cell lineage as vascular smooth muscle cells (VSMCs) but exhibit important differences with regard to their location in the vasculature, their morphology, and their function [10,11,12]. The mechanism by which PCs and ECs crosstalk to promote endothelial barrier function involves both cell–cell contacts [13] as well as soluble mediators, including growth factors, cytokines, and microRNAs (miRs) [14,15,16]. Reduced coverage of ECs with PCs can lead to vascular leakage and brain edema, as has been shown in several studies using viable PC-deficient mouse models [3,4]. Additionally, PC loss is a hallmark of many central nervous system disorders like Alzheimer’s disease, stroke, multiple sclerosis, trauma hemorrhagic shock and chronic obstructive pulmonary disease, to name a few [7,8,9,12,17,18]. Importantly, the migration of PCs away from the BBB is a critical manifestation of cerebral ischemia and correlates with capillary barrier breakdown, which is also observed in pathologies like sepsis or diabetic retinopathy [7,8,9,19].

The migration of PCs away from capillary ECs plays a critical role in the patho-physiology associated with capillary leakage, including BBB disruption, and is largely triggered by pro-inflammatory molecules [7,8,9]. Among pro-inflammatory cytokines, tumor necrosis factor-alpha (TNF-α) has been shown to be a prominent inducer of PC migration in vitro [10,20]. It is also a well-known mediator of vascular dysfunction, its upregulation under inflammatory conditions as well as in the plasma of ischemic stroke patients has been shown repeatedly and correlates with stroke damage and lesion size [21,22]. Importantly, increases in TNF-α have been observed in many patho-physiologies linked to the BBB [7,8,9,12,17,18,23,24,25,26,27,28,29,30]. Interestingly, the female sex steroid estrogen has been shown to promote microvascular stability and reduces the incidence of hemorrhagic strokes and cerebral aneurysms by preserving PC–EC interactions. Using EC–PC co-cultures, we have previously shown that estradiol (E2) preserved BBB integrity by inhibiting TNF-α-induced PC migration [31]. However, which mechanism(s) are involved in mediating this effect remains unclear.

Among TNF-α mechanism(s) of action, its regulatory effects on miR-expression levels play an important role in different cell types [32,33,34,35]. These short non-coding RNA molecules are important mediators for the regulation of gene expression and play major roles in a wide variety of diseases [36,37]. Changes in the miR-profile have shown to be associated with patho-physiologies leading to capillary leakage [38,39,40]. In this regard, increases in miR638 levels have been observed in trauma hemorrhagic shock [28], chronic obstructive pulmonary disease (COPD)/emphysematous lung destruction [41,42], acute stroke [43] and traumatic brain injury [44] as well as post-traumatic epileptogenesis [45]. Interestingly, miR638 is responsive (increases) to oxidative stress [46], which is known to disrupt the BBB [47]. Moreover, increased miR638 has been shown to induce metastasis in hepatocellular carcinoma by disrupting capillary barrier function [48]. TNF-α levels are reported to increase in patho-physiologies associated with capillary barrier/BBB disruption. In this regard, TNF-α levels increase following stroke [24,27], acute ischemic stroke [27], trauma [23], chronic neuroinflammation [26], hemorrhage, infection/sepsis [25], COPD, etc. Since TNF-α disrupts BBB integrity by stimulating PC migration [31], together with the fact that miR638 has been shown to influence cancer cell migration [49,50], it is feasible that TNF-α mediates its BBB disruptive actions by stimulating miR638. In the present study, we investigated the contribution of miR638 in mediating the pro-migratory actions of TNF-α in PCs.

Estrogens regulate miRs via both genomic as well as non-genomic mechanisms and have been intensively investigated in breast cancer [51,52]. The role of estrogen-regulated miRs in the cardiovascular system under physiological and pathological conditions has become an important area of research as well [53,54]. For example, it has been demonstrated that E2 promotes endothelial proliferation, migration and tube formation via the upregulation of miR126 [54]. In addition, E2 modulates VSMC migration and proliferation via regulation of miR221 and -193a [54]. Whether estrogens modulate miR638 expression and its mode of action remains unknown. Moreover, the role of estrogen receptor alpha (ER-α), estrogen receptor beta (ER-β) or G-protein coupled estrogen receptor (GPER) in mediating its actions was explored.

Stroke-induced BBB disruption is a leading cause of disability and mortality in women. Compared to pre-menopausal women, the overall risk of stroke is higher in post-menopausal women [55]. The fact that menopause is accompanied by a drop in estrogen levels suggests that it may induce protective actions. Consistent with this notion, the vascular protective actions of E2 on vascular structure and function are well established [56]. Experimental studies in animal models have clearly demonstrated the protective actions of E2 against ischemic stroke induced brain damage [57,58,59,60]. Interestingly, E2 counteracts the deleterious actions of TNF-α on vasculature [61,62] and pulmonary system [63] and abrogates its effects on vascular cells [62]. Importantly, we have recently shown that estrogen counteracts the TNF-α-induced migration of PCs and prevents its barrier-disrupting actions [31] via estrogen receptor-driven mechanisms. However, which TNF-α driven mechanism is targeted by estrogen remains unknown. Since both TNF-α and miR638 are increased in patho-physiologies associated with barrier disruption, we hypothesize that TNF-α stimulates PC migration by stimulating miR638. Moreover, E2 inhibits TNF-α-induced PC migration by inhibiting miR638 production.

To test the above hypothesis, in the present study, using molecular approaches in cultured PCs, we investigated whether TNF-α increases miR638 expression; miR638 mimics the effects of TNF-α on PC migration; pro-migratory effects of TNF-α in PCs are miR638-mediated; E2 modulates miR638 levels in PCs; E2 abrogates the effects of TNF-α on PC migration by counteracting miR638; the effects of E2 on miR638 are estrogen receptor-specific; the observed effects of estradiol are miR638-specific; miR638 triggers PC migration by activating MAPK and Akt signaling; actin in PCs migrating in response to TNF-α and miR638 is differently expressed in the presence and absence of E2.

## 2. Results

We have previously shown that estradiol (E2) abrogates the disruptive effects of TNF-α in BBB integrity by inhibiting TNF-α-induced PC migration. However, the underlying mechanism(s) involved remain unknown. Increasing evidence suggests that microRNAs (miRs) play a role in BBB integrity and can mediate the actions of both TNF-α and estrogen. Since increases in both miR638 and TNF-α have been observed in patho-physiologies (trauma, stroke, ischemia, COPD, etc.) associated with capillary barrier dysfunction linked to PC migration, we hypothesized and tested whether miR638 mediates the barrier-disrupting effects of TNF-α and whether this process is abrogated by E2.

### 2.1. Tumor Necrosis Factor Alpha (TNF-α) Induced Expression of miR638

**Figure 1:**
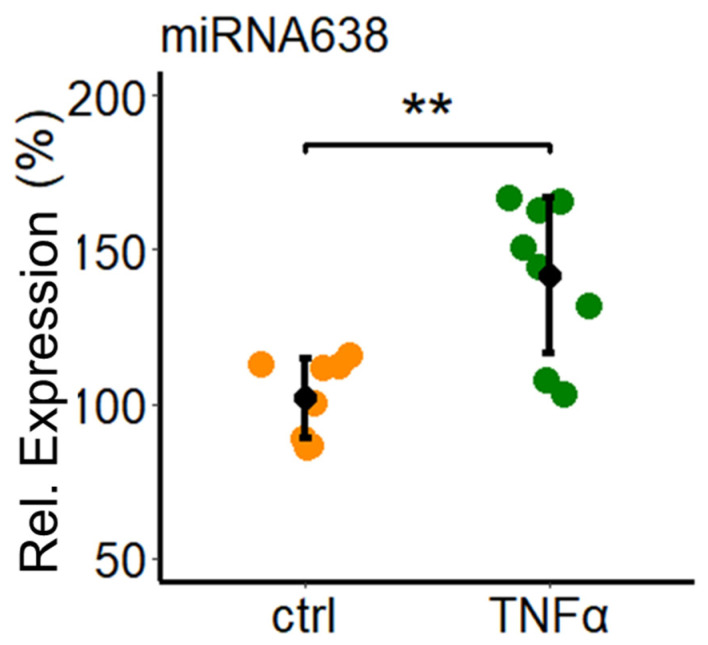
TNF-α upregulates the expression of miR638 in PCs. The sub-confluent monolayers of PCs were treated with TNF-α (10 ng/mL) alone or with a vehicle (ctrl) for 24 h. Subsequently, reverse transcription of isolated total RNA was performed and the expression levels of miR638 were quantified by PCR using the TaqMan miRNA assay kit. Expression levels were normalized to internal controls U44 and U48. Experiments were performed at least three times in triplicates or duplicates, and the data represent mean ± SD. ** *p* < 0.05, compared to control.

As a first step, we investigated whether TNF-α modulates miR638 in PCs. Treatment of PCs with TNF-α (10 ng/mL) for 24 h induced miR638 expression. As shown in Figure 1, compared to vehicle treated control, the expression of miR638 in response to TNF-α increased from 102 ± 13 to 142 ± 25 (*p* < 0.01; *n* = 8), respectively.

### 2.2. Transfection Efficiency miR638

**Figure 2:**
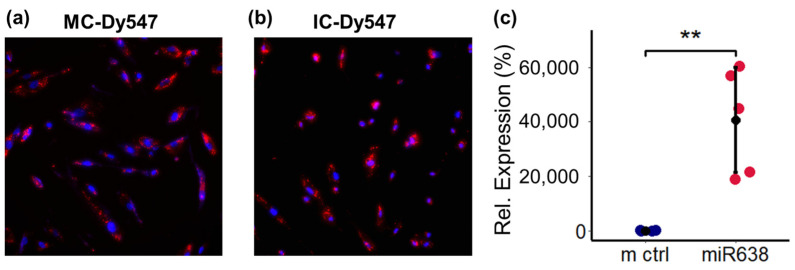
Transfection efficiency miR638 in PCs. Representative photomicrographs showing the transfection efficiency of miR638 mimic (**a**) and anti-miR (**b**) in PCs. Photomicrographs depict fluorescence microscopy images of PCs transfected with mimic (MC) or anti-miR (IC) Dy547. Red: mimic (MC) or anti-miR (IC) Dy547; blue: HOECHST 33342-transfected cells. Panel (**c**) depicts the transfection efficacy of miR638 in PCs using qRT-PCR. PCs were transfected with the vehicle (m ctrl) or with miR638 for 6 h and subsequently left for 24 h in normal medium prior to staining or sample extraction for qRT-PCR analysis. Experiments were performed at least three to five times to confirm efficient transfection. Values represent mean ± SD; *p* < 0.01 ** compared to control.

Next, we determined whether miR638 modulates PC migration. To achieve this and to assess whether miR638 mediates the effects of TNF-α on PC migration, we transfected PCs with miR638 mimics and/or its anti-miR (inhibitors) to increase or decrease miR levels, respectively. As a first step, we assessed the transfection efficiency of PCs after transfecting the cells with Dy547-labelled controls and through visualization with fluorescent microscopy (Figure 2a,b). Additionally, the levels of miR in the cells were determined by means of quantitative reverse transcription PCR after the transfection of the cells with the corresponding mimics (Figure 2c). Compared to the vehicle-treated control, the robust expression of miR638 was achieved with increase in relative expression from 100 ± 5.46 in the control to 40,605 ± 19,350 (mean ± SD; *p* < 0.01).

In order to rule out the possibility that the observed effects might be due to changes in cell viability, MTT assays as well as crystal violet staining (CVS) were performed in the miR638-transfected cells. No significant differences in cell viability were observed be-tween cells transfected with mimics or inhibitors for miR638 and scrambled controls (Supplementary Figure S1).

### 2.3. miR638 Induces PC Migration

**Figure 3:**
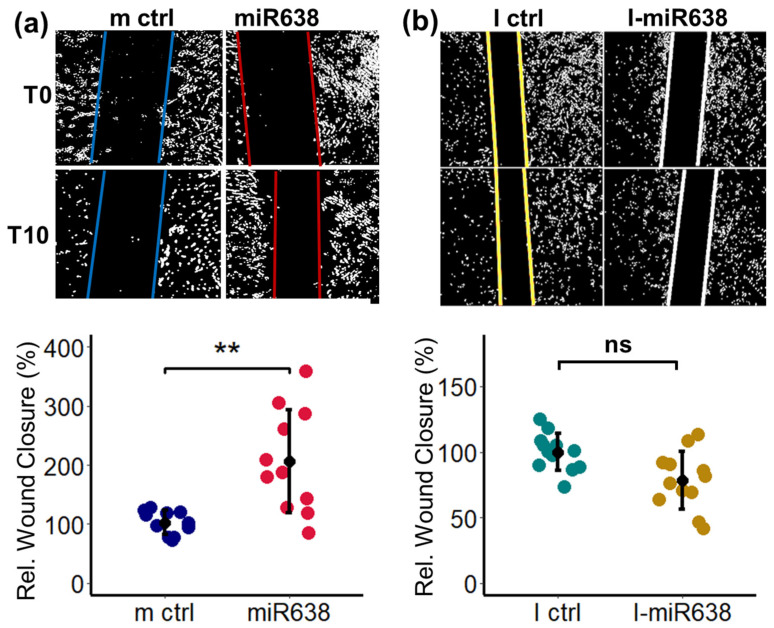
miR638 induces PC migration. PCs were transfected with miR638 or scrambled control (m ctrl) (**a**) and with miR638 inhibitor (I-miR638) (**b**). Cells were allowed to recover for 48 h before the scratch wound was induced and migration was assessed 10 h thereafter. Panel (**a**,**b**) also depict representative images of PC migration in response to miR638 at time (T) T0 and T10. Experiments were performed three times in triplicate or quadruplicate, and data represent mean ± SD; ns *p* > 0.05, *p* < 0.05 **.

After confirming the potency of our transfection method, we investigated the pro-migratory actions of miR638 in PCs by employing wound healing assays. In this context, scratch assays were performed 48 h after transfecting the PCs with mimics and inhibitors for miR638. As shown in Figure 3a, we observed a significant increase in wound closure in PCs transfected with miR638 mimics from 100% in mimic-control to 206% in PCs transfected with miR638 mimic. Furthermore, as shown in Figure 3b, the inhibition of miR638 slightly decreased migration but not significantly.

### 2.4. TNF-α Induces PC Migration via miR638

**Figure 4:**
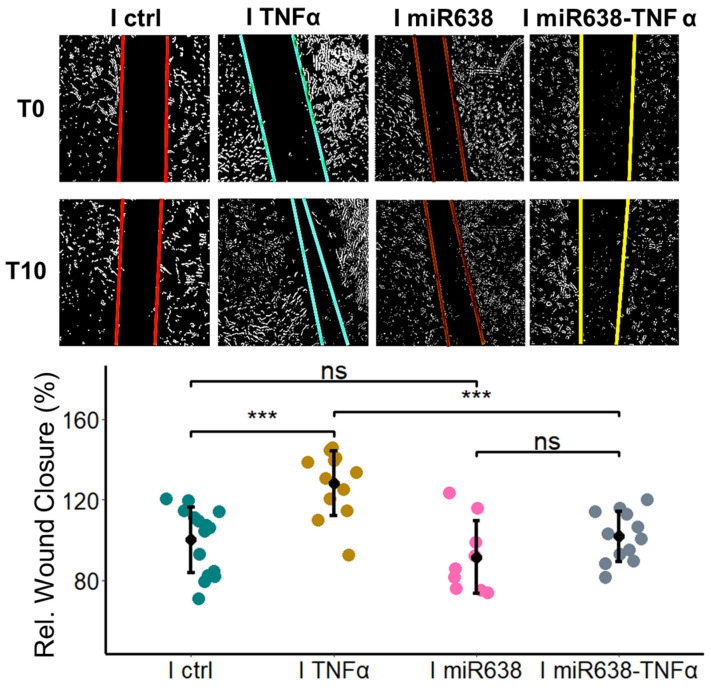
Inhibition of miR638 prevents TNF-α-induced migration of PCs. Cells were transfected with anti-miR (I miR638) or scrambled control (I ctrl) and left to recover for 48 h before a scratch-wound assay was performed in presence or absence of TNF-α (10 ng/mL). Wound closure was assessed after 10 h and representative contrast adjusted images (for clarity) are shown at T0 and T10. Experiments were performed at least three times with four or five replicates, and data represent mean ± SD. ns *p* > 0.05, *** *p* < 0.001.

Next, we confirmed whether miR638 mediates the pro-migratory actions of TNF-α in PCs. Briefly, the effect of TNF-α on PC migration was assessed in cells that were transfected with inhibitors for miR638. As shown in Figure 4, treatment with TNF-α induced migration in PCs transfected inhibitory miR control but not in PCs transfected with inhibitory miR638. TNF-α induced PC migration from 100 ± 16.4 in anti-miR control to 128 ± 16.1%, whereas in PCs transfected with inhibitory miR638, the treatment with TNF-α failed to induce migration.

### 2.5. Estradiol Downregulates the Basal and TNF-α-Induced Expression of miR638

**Figure 5:**
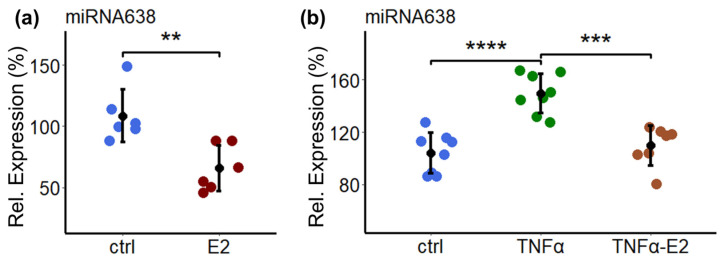
E2 downregulates basal as well as TNF-α-induced expression of miR638 in PCs. Cells were treated with E2 (10^−8^ M) for 24 h (**a**) and with TNF-α (10 ng/mL) alone or in combination with E2 (10^−8^ M) or vehicle (ctrl) for 24 h (**b**). The reverse transcription of isolated total RNA was performed and the expression levels of miR638 were assessed by PCR, using the TaqMan miRNA assay kit. Expression levels were normalized to internal controls U44 and U48. Experiments were performed at least three times in duplicates or triplicates, and the data represents mean ± SD. ** *p* < 0.05, *** *p* < 0.01, **** *p* < 0.001 compared to control or TNF-α.

Since we observed that miR638 mediates the pro-migratory actions of TNF-α in PCs, we further investigated whether E2 abrogates TNF-α-induced migration by downregulating miR638. As shown in Figure 5a, the treatment of PCs with 10^−8^ M E2 downregulated the expression of miR638 from 108 ± 21.6 in the vehicle-treated control to 65.6 ± 18.8 in PCs treated with E2, a decrease of 42% (*p* < 0.01). Moreover, E2 inhibited the stimulatory effects of TNF-α on miR638 expression. As depicted in Figure 5b, E2 lowered TNF-α-induced expression from 150 ± 15.1% to 110 ± 15.2% (*p* < 0.001).

### 2.6. Estradiol Downregulates miR638 via Estrogen Receptor Beta (ER-β)

**Figure 6:**
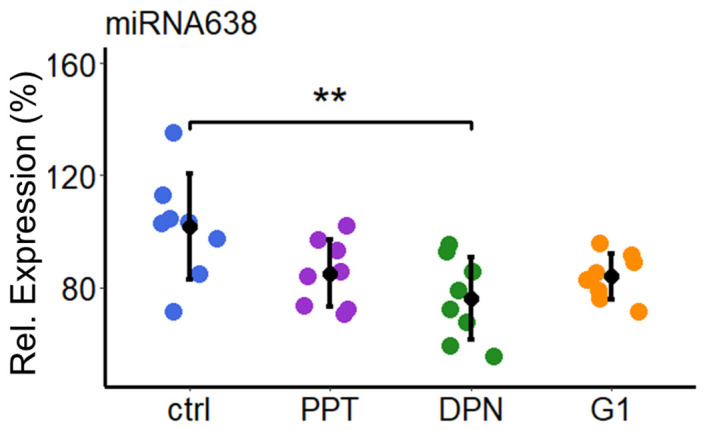
Estradiol downregulates the expression of miR638 in PCs via ER-beta. PCs were treated with the ER agonists PPT (ER-α), DPN (ER-β), and G-1 (GPER) (10^−7^ M) for 24 h before they were lysed for qRT-PCR. The reverse transcription of isolated total RNA was performed and the expression levels of miR638 were assessed through real-time PCR using the TaqMan miRNA assay kit. Expression was normalized to internal controls U44 and U48. Experiments were performed three times in triplicates or duplicates, and data represent mean ± SD. ** *p* < 0.01.

We further determined the involvement of estrogen receptors in mediating the effects of E2 on miR638 expression. We performed quantitative reverse transcription-PCR (qRT-PCR) in total RNA extracted from PCs treated with the three ER agonists: PPT (ER-α agonist), DPN (ER-β agonist), and G1 (GPER agonist). The expression levels of miR638 were significantly downregulated only by the specific ER-β agonist (by 21%, *p* < 0.01), while no statistically significant effects of ER-α and GPER agonists were observed (Figure 6).

### 2.7. TNF-α-Induced PC Migration Is Abrogated by the ER-β Agonist

**Figure 7:**
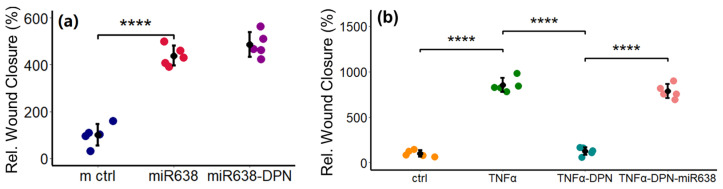
ER-beta inhibits TNF-α-induced PC migration by downregulating miR638 expression. Panel (**a**) depicts the modulatory effects of DPN on migration of PCs transfected with miR638. PCs transfected with miR638 were treated with or without DPN (10^−8^ M) and wound closure was assessed after 10 h. Panel (**b**) depicts the migration of PCs treated with TNF-α (10 ng/mL) in presence or absence of ER-β agonist DPN (10^−8^ M). DPN prevented TNF-α-stimulated PC migration. Panel (**a**) also shows that the inhibitory effects of DPN on TNF-α-induced PC migration are lost in PCs with the ectopic expression of miR638. PCs transfected with or without miR638 were allowed to recover for 12 h and subsequently the scratch-wound assay was performed in the presence or absence of TNF-α (10 ng/mL); TNF-α + DPN (10^−8^ M); or TNF-α + DPN (10^−8^ M) + miR638. Experiments were performed with a set of five for each treatment and the means of the three locations of a scratch were used to assess the changes. Data represent mean ± SD. **** *p* < 0.0001 compared to control, TNFα or TNFα + DPN.

Since miR638 expression was downregulated by ER-β agonist, we assessed its impact on TNF-α-induced PC migration. As shown in Figure 7a, the transfection of PCs with miR638 stimulated PC migration, and these effects were not blocked by the ER-β agonist DPN. Interestingly, co-treatment with DPN abrogated the stimulatory effects of TNF-α on PC migration (Figure 7b). Moreover, the ectopic expression of miR638 prevented the inhibitory effects of DPN on TNF-α-induced PC migration.

### 2.8. miR638 Induces ERK and Akt Phosphorylation

**Figure 8:**
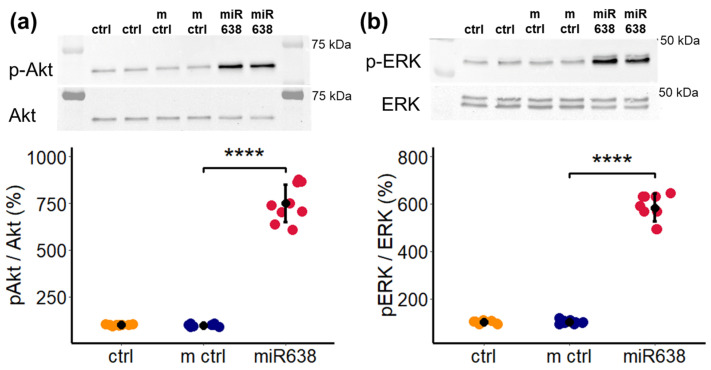
miR638 stimulates the phosphorylation of ERK and AKT kinases. Western blot analysis was performed on whole cell lysates of PCs, which were transfected with the miR mimic control (m ctrl) or miR638 or vehicle (ctrl) for 6 h. Representative Western blots of AKT phosphorylation at Serine residue 473 (**a**) and ERK phosphorylation at Threonine residue 202 and Tyrosine residue 204 (**b**) are shown above the graph. Total AKT and total ERK expression levels served as internal controls and were used for normalization. Experiments were performed three times in duplicates or triplicates, and data represent mean ± SD. **** *p* < 0.0001, compared to mimic–control (m ctrl).

We previously demonstrated that TNF-α activates both Akt and ERK phosphorylation. Since TNF-α induces its pro-migratory actions via miR638, we assessed whether it induces Akt and ERK phosphorylation. As shown in Figure 8a,b, transfection of PCs with miR638 significantly induced both Akt and ERK phosphorylation compared to mimic control.

### 2.9. ERK, but Not Akt, Mediates the Pro-Migratory Actions of miR638 in PCs

**Figure 9:**
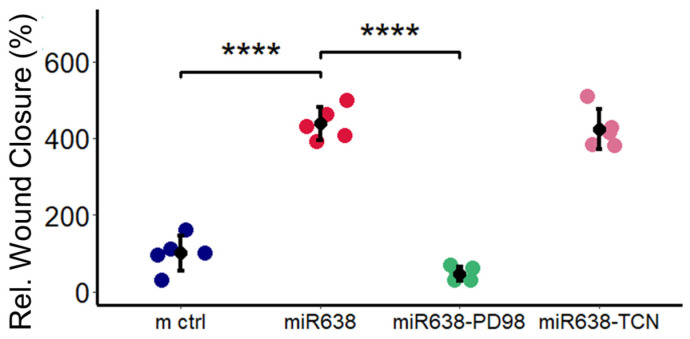
miR638 induces PC migration by inhibiting pERK but not pAKT. The inhibition of pERK by treating cells with the pharmacological inhibitor PD (10^−5^ M) during a 22 h post-transfection period (12 h recovery plus 10 h scratch period) prevented a miR638-induced increase in migration, whereas pretreatment with pAKT-inhibitor Triciribine (TCN, 1.5 × 10^−6^ M) showed no effect. Experiments were performed in a set of five for each treatment and means from three locations on a scratch were used to assess the changes. Data represent the mean ± SD. **** *p* < 0.0001 compared to m ctrl or miR638.

To assess the role of ERK and Akt in mediating the stimulatory actions of miR638 on PC migration, we assessed the migration of miR638 transfected PCs in presence and absence of both Akt and ERK inhibitors. As shown in Figure 9, the co-treatment of PCs with ERK phosphorylation inhibitor PD98059 abrogated miR638-induced PC migration, whereas the co-treatment of PCs with Akt inhibitor TCN failed to block the pro-migratory actions of miR638 in PCs, as assessed by wound closure assay. Compared to miR–control, miR638 induced migration from 100 ± 46.4% to 438 ± 46.4% (*p* < 0.008), and these stimulatory effects of miR638 were inhibited to 47 ± 18% (*p* < 0.008) in PCs co-treated with ERK inhibitor PD98059 but remained unaltered in PCs co-treated with Akt inhibitor TCN.

### 2.10. E2 Modulates TNF-α- and miR638-Induced Actin Dynamics in Migrating PCs and Prevents Filopodia-Like Structure

**Figure 10:**
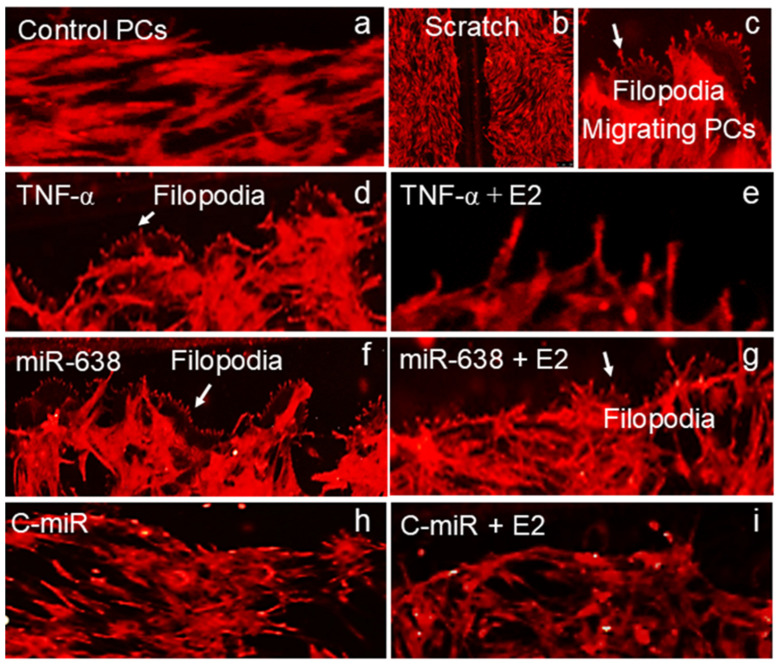
Estradiol inhibits TNF-α-induced PC migration by modulating F-actin dynamics. Representative photomicrographs depict fluorescent images of the ActinRed-555-stained migrating edge of PCs treated with TNF-α or transfected with miR638 in presence and absence of E2 (10^−8^ M). Panels (**a**–**c**) show representative photomicrographs of ActinRed-555-stained PCs, control (**a**), a scratch in PC monolayer (**b**) and filopodia (high magnification) in migrating cells (**c**). Structurally, compared to untreated control (**a**) PCs treated with TNF-α (**d**) as well as miR638 (**f**) but not control miR (C-miR, panel (**h**) showed filopodia-like structures in PCs on the outer edge of the scratch. Moreover, co-treatment with E2 prevented the formation of filopodia-like structures in response to TNF-α (**e**) but not miR638 (**g**). No filopodia-like structures were observed in PCs treated C-miR (**h**) and C-miR + E2 (**i**). All experiments were repeated three times with ten images for each scratch in each treatment group. White arrows indicate filopodia-like structures.

Changes in PC morphology and actin dynamics drive PC migration. To assess whether TNF-α and miR638 promote PC migration by modulating actin dynamics, we stained PCs with ActinRed 555 and assessed morphological changes in migrating PCs on the outer edge of the scratch. Representative photomicrographs in Figure 10a depict actin positive non-migrating PCs, whereas Figure 10b depicts a scratch in actin-stained PCs. Figure 10c shows filopodia-like structures (marked with white arrows) in migrating PCs at a higher magnification on the outer edge of the scratch. The qualitative assessment of PCs treated with TNF-α showed the presence of filopodia-like structures (white arrows) in the absence (Figure 10d) but not in the presence of E2 (Figure 10e). Similar to TNF-α, filopodia-like structures (white arrows) were present in PCs treated with miR638 (Figure 10f); however, in contrast to TNF-α, E2 did not modulate the structural effects of miR638 on PCs and the presence of filopodia-like structures Figure 10g). In PCs transfected with control–miR and treated with or without E2, no significant changes in actin or morphology were observed (Figure 10h,i).

### 2.11. Modulatory Effects of E2 on Basal Expression of Other Migration Influencing miRs in PCs

**Figure 11:**
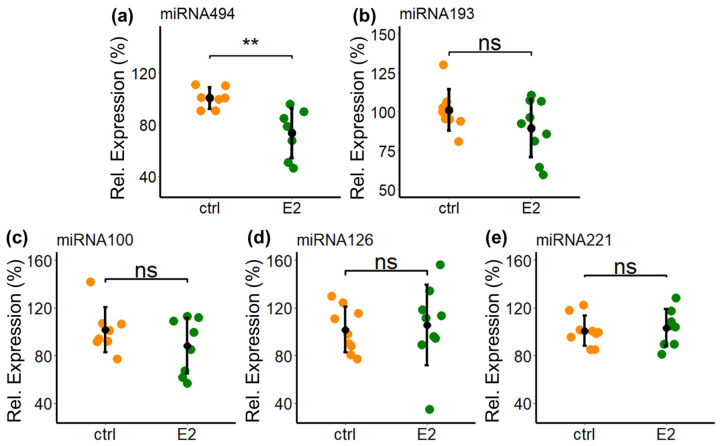
E2 on basal expression of other miRs in PCs. E2 downregulates the basal expression of miR494 (**a**) but not miR193 (**b**), miR100 (**c**), miR126 (**d**) and miR221 (**e**). PCs grown to sub-confluence were treated with E2 (10^−8^ M) for 24 h and subsequently lysed for qRT-PCR. Reverse transcription of isolated total RNA was performed and expression levels of various miRs were assessed by real-time PCR using the TaqMan miRNA assay kit. Expression was normalized to internal controls U44 and U48. Experiments were performed three times in triplicates or duplicates, and data represent mean ± SD. ** *p* < 0.01; ns—not significant.

Apart from miR638, other miRs can also influence migratory behavior in cells. Previous results from our lab have shown the regulation of several miRs in vascular smooth muscle cells (VSMCs) after E2 treatment [64]. As PCs are phenotypically similar to VSMCs, we screened their potential impact and involvement in PCs following E2 treatment. In this regard, we screened the effects of E2 on the expression of miR494, miR193a, miR100, miR126 and miR221. As shown in Figure 11, treatment with E2 significantly inhibited miR494 expression (inhibition by 24%, *p* < 0.05 vs. vehicle) but did not alter the expression of miR193a, miR100, miR126 and miR221.

### 2.12. E2 Inhibits TNF-α-Induced Expression on miR193a but Not miR494 in PCs

**Figure 12:**
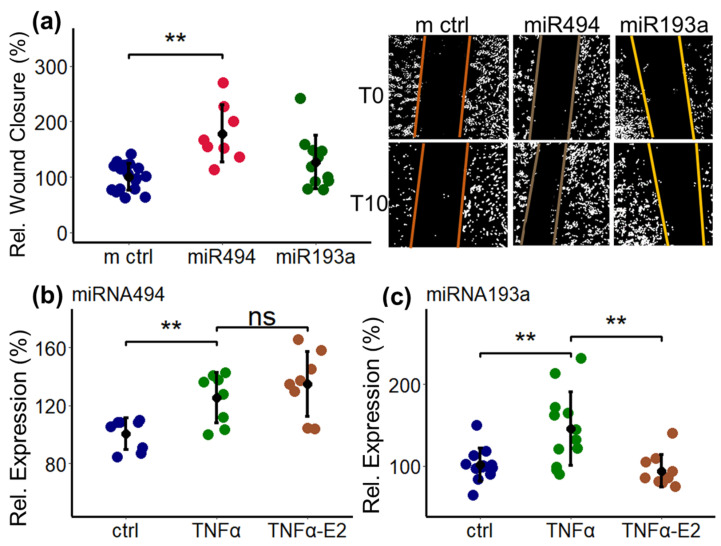
Effects of miR494 and miR193a on PC migration and the modulatory effects of E2 on their TNF-α-stimulated expression. Transfection with miR494 but not with miR193a induces PC migration (**a**). PCs were allowed to recover 48 h after transfection (with miR494 or miR193a) before the scratch wound was induced and migration was assessed 10 h afterwards. Panel (**a**) also depicts representative contrast adjusted images (for clarity) of PC migration in response to miR494 and miR193a at time (T) T0 and time T10. Panels (**b**,**c**) depict the modulatory effects of E2 on the TNF-α (10 ng/mL)-induced expression of miR494 and miR193a in PCs. Treatment with E2 (10^−8^ M) for 24 h did not modulate the TNF-α-induced expression of miR494 but abrogated its stimulatory actions on miR193a. Experiments were performed three times in triplicate or quadruplicate, and data represent mean ± SD; *p* < 0.05 **; ns—not significant.

To assess whether the inhibitory effects of E2 on PC migration may involve other TNF-α-stimulated miRs, we assessed the impact of miR193a and miR494 on PC migration. Transfection with miR494 induced PC migration (Figure 12a), whereas transfection with inhibitory miR494 decreased PC migration (Supplementary Figure S2). Interestingly, E2 had no effect on the TNF-α-induced expression of miR494 (Figure 12b) even though it downregulated the basal expression of miR494 (Figure 11a), suggesting that miR494 may have a role in regulating basal but not the TNF-α-induced migration of PCs. With regard to miR193a, transfection with miR193a did not change PC migration as compared to miR controls (Figure 12a). However, E2 abrogated the TNF-α-induced expression of miR193a (Figure 12c), suggesting that this miR may have a role in regulating activities other than PC migration.

## 3. Discussion

We previously reported that estrogen mediates its protective effects on the BBB by targeting PCs within the neurovascular unit and preventing their migration away from ECs in response to potent pro-inflammatory cytokines known to disrupt barrier integrity [31]. Although we demonstrated the abrogatory effects of E2 on TNF-α-induced EC–PC barrier disruption and PC migration, the underlying mechanisms remained unclear. As an extension of our previous findings, here we demonstrate that E2 mediates its protective actions by inhibiting the TNF-α-induced migration of PCs by targeting miR638. We provide the first direct evidence that TNF-α induces PC migration by stimulating miR638. Moreover, E2 inhibits PC migration by blocking the basal and TNF-α-stimulated expression of miR638 and that these inhibitory actions of E2 are mediated via ER-β and involve MAPK, but not Akt, phosphorylation. Our findings demonstrate a potential mechanism of estrogen’s neuroprotective action by abrogating TNF-α-induced PC migration via ER-β/miR638/MAPK-P axis.

Pro-inflammatory cytokines, including TNF-α, are known to damage/disrupt the BBB and are associated with neurodegenerative disorders [10,20]. Moreover, changes in miRs have been documented in various neurodegenerative disorders [65]. However, their specific role in neurodegenerative etiology remains unclear. Our findings that TNF-α stimulates miR638 levels in PCs; miR638 induces PC migration; and miR638 anti-miRs abrogates TNF-α-induced PC migration, together with our previous finding that TNF-α disrupts the BBB [31], provide the first evidence that TNF-α potentially mediates its BBB disruptive actions via miR638. This contention is further supported by the findings that increases in miR638 as well as TNF-α have been observed in multiple patho-physiologies involving capillary leakage including BBB disruption [10,20,21,22,23,24,25,26,27,28,41,42,43,44,45]. However, direct evidence for this association needs to be confirmed in an in vivo setting.

The increased incidence of stroke and neurodegenerative disorders in women following menopause [55] suggests that estrogen may protect BBB integrity. Animal studies have shown that estrogen protects against ischemia and trauma-induced brain damage [57,58,59,60]. Although many studies have looked at the impact of E2 in brain capillary ECs, very little is known about its impact on capillary PCs. Based on the importance of capillary PCs in maintaining BBB integrity, we previously demonstrated that human brain capillary PCs express ERs and that estrogen protects EC–PC barrier integrity by preventing their migration [31]). Moreover, we demonstrated that E2 prevents TNF-α-induced PC migration [31]. Interestingly, E2 has been shown to facilitate crosstalk between ECs and PCs by regulating platelet-derived growth factor B (PDGF-B) [66], which drives PC recruitment and promotes vessel stabilization. In line with the above findings, here we provide the first evidence the E2 prevents PC migration by abrogating TNF-α-induced miR638 production.

The regulation of miR expression levels is a well-known mechanism in mediating the physiological but also pathological actions of TNF-α, as well as E2 [32,33,34,35,51,52,53,54]. Since in-creases in miR638 as well as TNF-α levels have been observed in multiple patho-physiologies associated with capillary leakage [7,8,9], together with our observation that E2 inhibits TNF-α-induced PC migration [31], we hypothesized that E2 inhibits PC migration by downregulating the TNF-α-induced expression levels of miR638. In support of this hypothesis, we demonstrate that E2 inhibits both basal and TNF-α-induced miR638 expression. Moreover, increasing the intracellular levels of miR638 in PCs significantly enhanced cell migration, while the repression of miR638 levels in PCs by transfecting the cells with miR inhibitors did not show a significant opposing effect on migration. This finding can be explained by the low basal miR638 expression levels in PCs, which is visible by the high CT values obtained in quantitative reverse transcription PCR experiments. The downregulation of miR638 levels, however, mimicked the effect of E2 and prevented the increased migration of PCs after TNF-α stimulation. This supports our hypothesis that the inhibitory effects of E2 on miR638 expression are responsible for counteracting the TNF-α-induced migration.

To assess the specificity of E2 in targeting miR638 to inhibit TNF-α-induced migration of PCs, we tested its impact on miR494 and miR193a. We found that in PCs, TNF-α induced both the miR494 and miR193a levels; E2 inhibited the TNF-α-induced levels of miR193a, but not miR494; and miR193a did not stimulate PC migration, suggesting that E2 specifically blocks TNF-α-induced PC migration by targeting miR638. The fact that miR494 stimulated PC migration but E2 did not abrogate the stimulatory effects of TNF-α on miR494 suggests that it may play a role in PC migration in basal, but not inflammatory conditions linked to increases in TNF-α.

Very little is known regarding the effects of miR638 in PCs. Results obtained in studies using other cell types are divergent with respect to the function of miR638. Many studies have noted the upregulated levels of miR638 in different cancer types such as melanoma, breast cancer, renal carcinoma and adrenocortical carcinoma [48,49,50,66]. The oncogenic properties of miR638 in the above-mentioned, but also other, cancers include the downregulation of tumor suppressor genes like PTEN, p53 or BRCA1 [67,68,69,70]. On the other hand, several studies also found the suppressive effects of miR638 on the invasiveness of tumor cells by targeting tetraspanin-1 or phospholipase-C, or by regulating the wnt/b-catenin pathway [67,71,72,73,74]. In contrast to the relatively low expression levels of miR638 that we observed in PCs, Li et al. noted a high expression of the same miR in SMCs, which was responsible for antagonizing the PDGF-BB-induced proliferation and migration [75]. With regard to the TNF-α or E2 regulation of miR638, the only studies conducted so far were performed in TNF-α-treated human umbilical cord ECs (HUVECs) and showed a decrease in miR638 upon cytokine stimulation [35,76], while in the present study, we observed a stimulatory effect on miR638 expression by TNF-α. However, miR expression levels and functions are highly cell type-specific, and dual roles of miRs in different cell types have been shown many times before [77,78]. For instance, while E2 downregulates miR221 and miR193a in HUVECs as well as VSMCs, the impact of these miRs on cell proliferation and migration in the respective cell types is exactly the opposite [64]. Mechanisms responsible for these differential effects are beyond the scope of this study, but a highly plausible explanation might lie in the abundance of target mRNA molecules in different cell types. Moreover, one miR can affect up to hundreds of mRNA molecules and one mRNA can again be targeted by several different miRs, which further contributes to the plausibility of above-mentioned contradictory observations [79]. Since neighboring miR binding sites on an mRNA can also impact responses and lead to the cooperative miR silencing positions of miR binding sites can also affect the extent of observed responses [80].

Estrogens regulate miRs via both genomic as well as non-genomic mechanisms and have been intensively investigated in breast cancer [51,81]. The role of estrogen-regulated miRs in the cardiovascular system under physiological and pathological conditions has become an important area of research as well [54]. It has been demonstrated, for example, that E2 promotes endothelial proliferation, migration and tube formation via the upregulation of miR126 [77]. In addition, E2 modulates VSMC migration and proliferation via the regulation of miRNA221 and miRNA193a [64]. Whether estrogens modulate miR638 expression and its mode of action has not been investigated. Our finding that miR638 expression was significantly downregulated by ER-β agonist DPN, but not by ER-α and GPER agonists, MPP and G1, respectively, implicates that ER-β mediates the inhibitory actions of E2 on PC migration. This role of miR638 is further confirmed by our finding that DPN blocks TNF-α-induced PC migration, and this effect is neutralized by the ectopic expression of miR638. This highlights the specificity and importance of ER-β in abrogating the stimulatory effect of TNF-α on PC migration by downregulating miR638. To the best of our knowledge, this is the first report showing that E2 downregulates the basal as well as TNF-α-induced expression of miR638 in PCs and inhibits PC migration by lowering miR638. Importantly, these effects are mediated via ER-β, but not GPER, thereby implicating the genomic mechanism. Interestingly, ER-β plays a key role in preventing growth/metastasis in breast cancer cells [81] as well as inhibiting the migration of VSMCs, which are phenotypically similar to PCs [82].

With regard to the molecular mechanism, we have previously shown that, even though E2 inhibits ERK1/2 and Akt phosphorylation, it inhibits TNF-α-induced migration solely by repressing ERK1/2 phosphorylation [31]. Consistent with our previous observation with TNF-α, the transfection of PCs with miR638 induced both ERK1/2 and Akt phosphorylation. Moreover, miR638-induced PC migration was blocked by the ERK1/2 inhibitor PD98059 but not by the Akt phosphorylation inhibitor Triciribine (TCN). These observations, together with our finding that TNF-α induces PC migration by increasing miR638, suggest that TNF-α mediates its pro-migratory actions in PCs by ERK1/2-*p* activation via miR638. Increased MAPK-phosphorylation has also been shown in another in vitro study to induce migration in PCs [83]. Furthermore, Takata et al. showed that TNF-α induces PC migration via MMP-9 release and that MMP-9 expression is increased via the activation of the PI3K/AKT cascade and ERK pathways [84]. Whether there is a link between AKT-/ERK-phosphorylation and induced PC migration remains unknown and beyond the scope of the present study. While estrogen signaling is associated with the increased phosphorylation of ERK and AKT in ECs, thereby promoting migration and proliferation, its action on these kinase cascades in VSMC is just the opposite, with a resulting decrease in mitogenic functions. Since PCs are phenotypically similar to VSMCs and seem to stem from the same cell lineage, our results fall in line with the previously mentioned observation of a decreased activity of these kinase cascades upon E2 treatment in PCs.

Which other mechanisms beyond ERK1/2 may mediate the pro-migratory actions of miR638 in PCs can only be speculated. Recent studies provide evidence that serum response factor (SRF) controls PC migration [85]. Moreover, SRF is a master regulator of actin cytoskeleton [86,87], which plays an active role in PC contractility and migration. Our finding that TNF-α and miR638 induced PC migration was associated with structural changes in actin distribution in migrating PCs, and these effects were absent in the presence of E2; it is feasible that TNF-α induces PC-migration by activating SRF and actin via miR638 and that E2 counteracts this action by inhibiting the TNF-α-induced expression of miR638. Interestingly, SRF acts as a master regulator for actin cytoskeleton and the signaling involves ERK1/2 activation, thereby adding further credence to an active role of SRF-actin-ERK1/2 axis in mediating miR638 actions on PC migration. However, in-depth studies are required to further investigate this possibility.

Determining the involvement of ER subtypes is an important step for therapeutics development, since considerable differences exist with regard to function between the three subtypes and ER specificity in different tissues [88]. For example, adverse effects of estrogen replacement therapy are observed regarding estrogen’s growth-promoting effect on breast and uterus via ER-α signaling. We therefore elucidated the involvement of ERs in mediating estrogens inhibitory effects on PC migration and found ER-β to be responsible for the downregulation of miR638 expression by E2 as well as for inhibiting TNF-α-induced migration. Importantly, ER-β signaling is also responsible for counteracting many other TNF-α-induced cellular changes by estrogen. In this manner, ER-β activation inhibits the inflammatory stimuli of TNF-α in SMCs [82], reduces the TNF-α-induced apoptosis of HUVECs and contributes to BBB protection following cytokine treatment and ischemic injury [89]. Our finding contributes to this already established significance of ER-β in mediating anti-inflammatory effects of E2. In addition, ischemic neuroprotection is mediated by ER-β activation, while hippocampal ER-β silencing increases neural inflammasome activation [90]. It is interesting to note that the preponderance of ER-β over ER-α has been shown to occur under different inflammatory as well as under hypoxic conditions, which might contribute to above-noted observations that ER-β signaling is more strongly involved in the anti-inflammatory signaling of estrogen compared to ER-α. Furthermore, ER-β has been associated with the selective regulation of MAPK pathways, while ER-α signaling primarily affected AKT cascades [91].

The relevance of our finding that E2 prevents the TNF-α-induced migration of brain capillary PCs may not be limited to the BBB. This contention is supported by the fact that capillary leakage is observed in multiple pathologies such as COPD, retinopathy, coats disease, and systemic infections [7,92,93]. Moreover, abnormalities in capillary PC coverage as well as increase in TNF-α and miR638 have also been documented [92,94]. Taken together, molecules which lower or counteract miR638 may be protective in patho-physiologies associated with capillary leakage. Although our findings demonstrate that E2 prevents TNF-α-induced PC migration by downregulating miR638 levels, other mechanisms may also be involved. For example, E2 has been shown to lower TNF-α levels [61] and counteract free-radical/oxidative stress-induced damage [60]. Future in-depth studies for the interactive role of E2, TNF-α and miR638 in regulating PC health may provide a better understanding of capillary integrity in various pathologies.

Study limitations: The findings of this study are solely derived from in vitro experiments conducted in cultured PCs. Although the results provide important insights on the role of miR638 in mediating the deleterious actions of TNF-α on BBB integrity and on the protective actions of E2, future experiments using PC–EC co-cultures as well as in vivo BBB integrity models are necessary to confirm whether E2 prevents TNF-α-induced barrier disruption by inhibiting miR638.

## 4. Materials and Methods

### 4.1. Cell Culture

hBVPs: human brain vascular pericytes (HBVPs, ScienCell, Carlsbad, CA, USA) between the 4-10th passage were cultured in Poly-L-Lysine (PLL)-coated flasks (2 µg/cm^2^) under standard tissue culture conditions (37 °C, 5% CO_2_) in growing media consisting of DMEM/F12 supplemented with antibiotic–antimycotic solution (AA: 100 μg/mL streptomycin, 100 μg/mL penicillin, and 0.025 μg/mL amphotericin B), GlutaMAX (1×), and 10% FBS. Media were changed every two or three days until sub-confluency.

### 4.2. Migration Studies

The migration of cells was assessed by a scratch-/wound-closure assay. Cells were plated in PLL-coated, 24-well plates and grown to confluence. Cells were starved overnight in starving media (DMEM/F12 supplemented with AA (100 μg/mL streptomycin, 100 μg/mL penicillin, and 0.025 μg/mL amphotericin B), GlutaMAX (1×), and 0.5% (sf.) FCS). If required, cells were pre-treated with antagonists/inhibitors for the assigned period of time, before the monolayers were scratched with a yellow pipette tip. Cells were washed once with HBSS (+Mg^2+^ +Ca^2+^) to remove loose cells, and treatment or vehicle was added. Images of each scratch were taken with an automated Olympus IX81 microscope (Olympus, Volketswil, Swizerland) at 0 h and at 10 h after treatment. The area of wound closure was determined using the software ImageJ (version 1.54f), and relative wound closure was calculated as follows: (area (T0) − area (T10))/area (T0). A schematic representation of the experimental set-up has been described before [31] and is provided in Supplementary Figure S3.

### 4.3. MicroRNA Expression Analysis

For miR expression analysis, cells were grown in 35 mm dishes and treated as specified before media were removed and cells were lysed by directly adding 300 μL RNA lysis buffer to the dish. Cell lysates were collected by scraping and samples were frozen at −80 °C until further processing. Total RNA was extracted using Quick-RNA MiniPrep kit from ZymoResearch, according to manufacturer’s protocol (by using a 5417R Centrifuge, Eppendorf, Hamburg, Germany). The concentration of purified total RNA was determined right after isolation by using a Tecan Spectrofluorometer reader (Infinite 200 NanoQuant, Tecan, Salzburg, Austria). RNA purity was checked by calculating the ratio of absorbance at 260/280 nm (>2.0) and 260/230 nm (>1.8).

### 4.4. Quantitative Reverse Transcription (qRT)-PCR

The analysis of miR abundance was performed by quantitative reverse transcription PCR with TaqMan miRNA assays. First, single-stranded cDNA was produced from 10 ng total RNA with the TaqMan miRNA Reverse Transcription Kit in a reaction volume of 15 μL containing deox-ynucleotide (dNTP) mix (1 mM), MultiScribe Reverse Transcriptase (50 U), Reverse Transcription Buffer (1×), RNase Inhibitor (0.3 U), and miRNA-specific RT primers (1×). cDNA synthesis was performed by incubation at 16 °C for 30 min, followed for 30 min at 42 °C and an inactivation step at 85 °C for 5 min. cDNA was stored at −80 °C until further processing. After the addition of 75 μL of nuclease-free water, the amplification and detection of specific miRNAs was performed using a Bio-Rad CFX Real-Time PCR Detection System. PCR reaction volume was 10 μL including 5 μL 2× TaqMan Fast Advanced Master Mix, 0.5 μL nuclease-free water, 0.5 μL 20× TaqMan miRNA Assay Mix, and 4 μL diluted cDNA. Incubation for 2 min at 50 °C and 20 s at 95 °C was followed by 40 cycles of 95 °C for 3 s and 60 °C for 30 s. For data normalization, RNU44, RNU48, RNU49, and/or RNU6b were used as internal controls. Relative gene expression was calculated using the 2^−ΔΔCt^ method. MicroRNA sequencies and primers were as follows: hsa-miR-638 5′-AGGGAUCGCGGGCGGGUGGCGGCCU-3′; hsa-miR-100 5′-AACCCGUAGAUCCGAACUUGUG-3′; hsa-miR-193a-3p 5′-AACUGGCCUACAAAGUCCCAGU-3′; hsa-miR-494 5′-UGAAACAUACACGGGAAACCUC-3′; hsa-miR-221-3p 5′-AGCUACAUUGUCUGCUGGGUUUC-3′; and has-miR-126 5′-UCGUACCGUGAGUAAUAAUGCG-3′.

### 4.5. Transfection with MicroRNA Mimics and Inhibitors

PCs were cultured in growing media and transfection was carried out 48 h after plating. Lipofectamine2000 (A) and miR mimics/anti-miRs (B) were diluted in DMEM/F12 according to Table 1 and these mixtures were incubated 5 min at RT, before equal amounts of A and B were mixed together and further incubated for 20 min at RT. In the meantime, cells were washed 2× with HBSS (+Ca^2+^ +Mg^2+^) and transfection media (DMEM/F12 supplemented with GlutaMAX (2×) and 0.5% FBS), in an amount according to Table 1, was added. The miR oligonucleotides–Lipofectamine complexes were then added to each well. After 6h, transfection media was exchanged with normally growing or starving media, depending on the experiment.

To determine the effect of miRs on cell migration, PCs transfected with miRs for 6 h, as described above, were allowed to recover for 12 h to 48 h before performing the scratch assay. To assess the modulatory actions of various treatments (TNF-α, DPN, ERK1/2 inhibitor, and Akt inhibitor) on miR-induced PC migration, cells were treated with the specific modulators during the post-transfection recovery period and prior to the scratch.

We used 10 ng/mL of TNF-α to assess its modulatory actions on miR638 production and PC migration. Using concentration response curves, we have previously shown that this concentration optimally induces PC migration and disrupts endothelial barrier integrity without any toxic side effects [31].

### 4.6. Transfection Efficiency

Fluorescently labeled mimics or inhibitors were used to determine transfection efficiency using fluorescent microscopy. Cells were transfected as described above with Dy547-labeled mimics and inhibitors and subsequently, cell nuclei were stained with Hoechst33342 (0.5 μg/mL) for 30 min. Transfection efficiency was assessed microscopically. Additionally, qRT-PCR was performed after cells were transfected with mimics or scrambled control. At 24 h after transfection, cells were lysed in RNA lysis buffer and relative RNA concentration was assessed as described in the section on microRNA expression analysis.

### 4.7. Viability Studies

MTT test: hBVPs were plated in a 48-well plate and were allowed to recover for 48 h. Cells were transfected with mimics or inhibitors or scrambled controls, as described above, and left to recover for 48 h before MTT was added to a final concentration of 0.5 mg/mL. After 3 h, MTT media were removed and the cells were lysed in 100 μL DMSO by shaking for 5 min at RT. Absorbance was measured in a 96-well plate at 505 nm using a Tecan Spectrofluorometer reader (Tecan, Salzburg, Austria).

CVS staining: hBVPs were plated in a 48-well plate and allowed to recover for 48 h. Cells were transfected with mimics or inhibitors or scrambled controls, as described above, and left to recover for 48 h. Paraformaldehyde (PFA, 4%) was then added to the media (1:1) for 2 min, before media were removed and fresh PFA (4%) was added for 15 min at RT without shaking. Subsequently, cells were washed once with HBSS (+Mg^2+^ +Ca^2+^) and twice with ddH_2_O for at least 3 min each wash. Crystal violet staining (CVS) solution (0.5% aqueous solution in 25% methanol) was added for 10 min at RT while shaking slightly. CVS solution was then carefully removed, and cells were washed extensively with ddH2O until no water coloring was visible anymore. The solubilization of the cells was achieved by adding 1% SDS aqueous solution while shaking at RT for 5 min. The solution was transferred to a 96-well plate for reading the absorbance at 595 nm using a Tecan Spectrofluorometer reader (Tecan, Salzburg, Austria).

### 4.8. Immunofluorescence Labeling

Scratch assay was performed in cultured PCs treated with TNF-α or transfected for 6 h with miR638 or control–miR. After making a scratch, the cells treated with TNF-α or transfected with miRs were exposed to E2 (10^−8^ M) or vehicle for 10 h. Subsequently, the medium was aspirated and cells were washed with PBS. The cells were fixed with 4% paraformaldehyde (Sigma-Aldrich, St. Louis, MO, USA) for 15 min and washed twice with PBS, followed by two 5 min washes in PBS at room temperature. Samples were permeabilized with 0.1% Triton X-100 for 5 min and blocked with 5% bovine serum albumin (BSA) for 2 h at room temperature or overnight at 4 °C. Subsequently, samples were washed twice with PBS, followed by two 5 min washes in PBS and 30 min PBS at room temperature. Fluorescently labeled Phalloidin (ActinRed 555 ReadyProbes; R37112; Life Technologies, Eugene, OR, USA) was applied for 10 min at room temperature, following the manufacturer’s instructions, and washed twice in PBS, followed by two 5 min washes in PBS. Images of migrating cells stained with ActinRed 555 were taken using a fluorescent microscope (Olympus IX81 microscope, Olympus, Volketswil, Switzerland).

### 4.9. Statistical Analysis

Experiments were performed at least three times and data are represented as means ± SD unless stated otherwise. Statistical evaluation was performed by using R (version 4.4.0). If ANOVA assumptions were met, parametric testing was performed using one-way ANOVA and subsequent Tukey’s HSD multiple pairwise comparisons. If either one of the ANOVA assumptions were not met, non-parametric testing was performed using the Kruskal–Wallis rank sum test and subsequent pairwise Wilcoxon tests with Benjamini–Hochberg corrections for multiple comparisons.

## 5. Conclusions

In conclusion, the present study contributes to a better mechanistic understanding of estrogen’s well-known anti-inflammatory and neuroprotective actions. Our findings postulate that estrogen receptor β signaling might lead to reduced PC detachment from the vessel wall in response to inflammatory stimuli by the downregulation of cytokine (TNF-α)-induced miRNA638 expression, thereby protecting BBB integrity and preventing a cascade of events leading to the further aggravation of the condition.

## Figures and Tables

**Table 1:** Guidelines for diluting and preparing reagents from the transfection protocol for a final miR–mimic or miR–inhibitor concentration of 25 nM.

		35 mm Dish/ 6-Well Plate	24-Well Plate/ 48-Well Plate
A	Lipofectamine2000	2 μL	0.5 μL
	Media	98 μL	24.5 μL
B	Mimic or inhibitor (10 μM)	3 μL	0.75 μL
	Media	97 μL	24.25 μL
	Media on cells	1000 μL	250 μL

## Data Availability

All data supporting the findings of this study are available within the article and its supplemental data files or from the corresponding author upon reasonable request.

## Supplementary Materials

The following supporting information can be downloaded at: https://www.mdpi.com/article/10.3390/ijms252111416/s1.
