# Supplementary material for: 17β-Estradiol Abrogates TNF-α-Induced Human Brain Vascular Pericyte Migration by Downregulating miR-638 via ER-β
Source: Int J Mol Sci. 2024 Oct 24;25(21):11416. doi: 10.3390/ijms252111416 (PMC11547073; doi:10.3390/ijms252111416)
Supplement: Supplementary file 1 [file ijms-25-11416-s001.zip › ijms-3243169-supplementary.pdf]

Supplementary Materials

17β-Estradiol Abrogates TNF-α-Induced Human Brain Vascular Pericyte Migration by Downregulating miR-638 via ER-β

Lisa Kurmann [1], Giovanna Azzarito [1], Brigitte Leeners [1], Marinella Rosselli [1] and Raghvendra K. Dubey [1,2,*]

1 Department of Reproductive Endocrinology, University Hospital Zurich, 8952 Schlieren, Switzerland.
2 Department of Pharmacology & Chemical Biology, University of Pittsburgh, Pittsburgh, PA 15219, USA

kurmann_lisa@gmx.ch (L.K.); giovanna.azzarito@usz.ch (G.A.); brigitte.leeners@usz.ch (B.L.); marinella.rosselli@usz.ch (M.R.)

* Correspondence: raghvendra.dubey@usz.ch

Figure S1

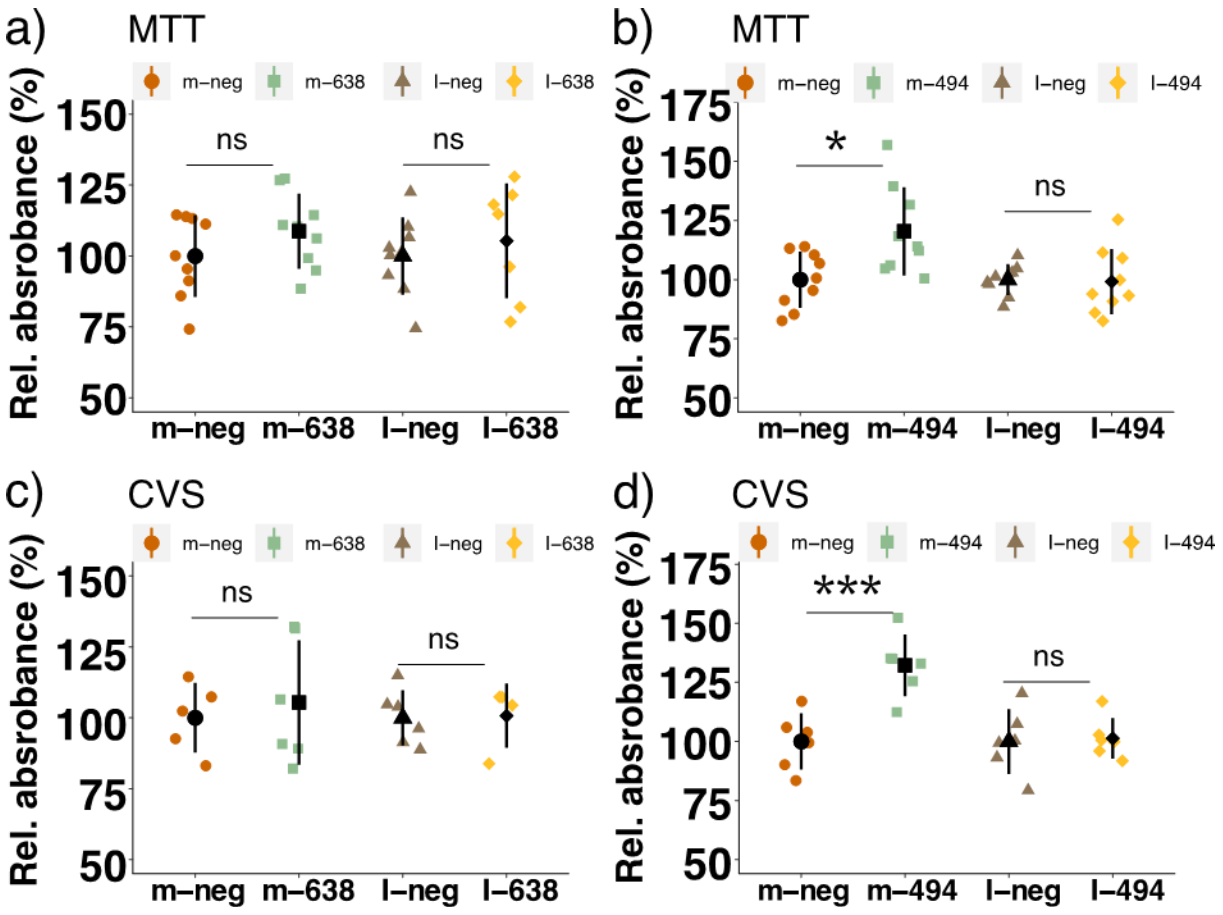

Figure S1: Effects of micro-RNA -494 and -638 on PC growth. Micro-RNA (m) 494 but not m-638 induces mitogenic actions in PCs. Graphs showing the effects of micro-RNA -638 (m-638), micro-RNA-494 (m-494) mimics and anti-miRs (I-638 or I-494) on PC viability as measured by Thiazol Blue Tetrazolium Bromide (MTT; **panels a and b**) and Crystal Violet Staining (CVS; **panels c and d**) assays. Significant increase in MTT and CVS staining was observed in PCs transfected with miR-494 mimic suggesting it may have pro-growth actions. Data represents mean +/- SD from 3 experiments, * $p<0.05$; *** $p<0.001$, ns not significant $p>0.05$, as compared to miR or I-miR controls (m-neg and I-neg).

Figure S2

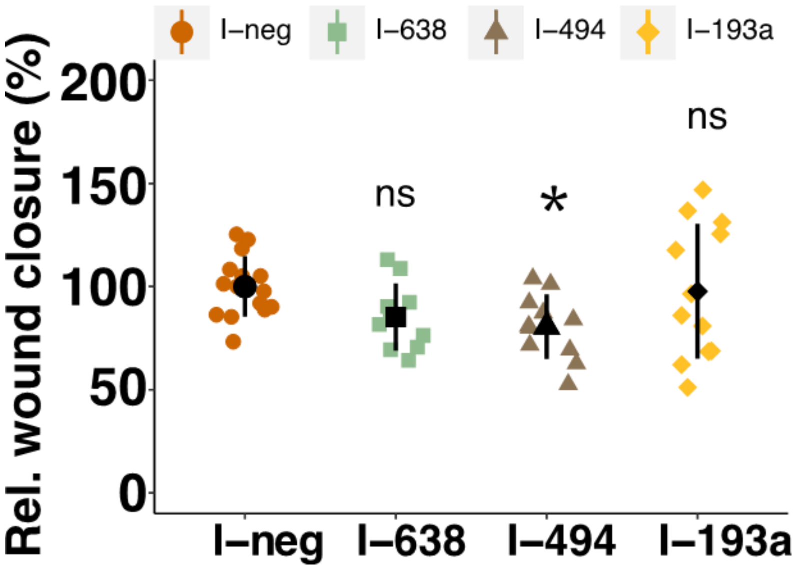

Figure S2 Modulatory effects of anti-miRs of miR-638, miR-494 and miR-193a PC migration. Cells were transfected with the respective anti-miR (I-638, I-494, I-193a) or scrambled control (I-neg) and let to recover for 48h before a scratch-wound assay was performed. Wound closure was assessed after 10h. Experiments were performed at least 3 times in 4 or 5 replicates and data represent mean +/- SD. ns $p > 0.05$, * $p < 0.05$.

Figure S3.

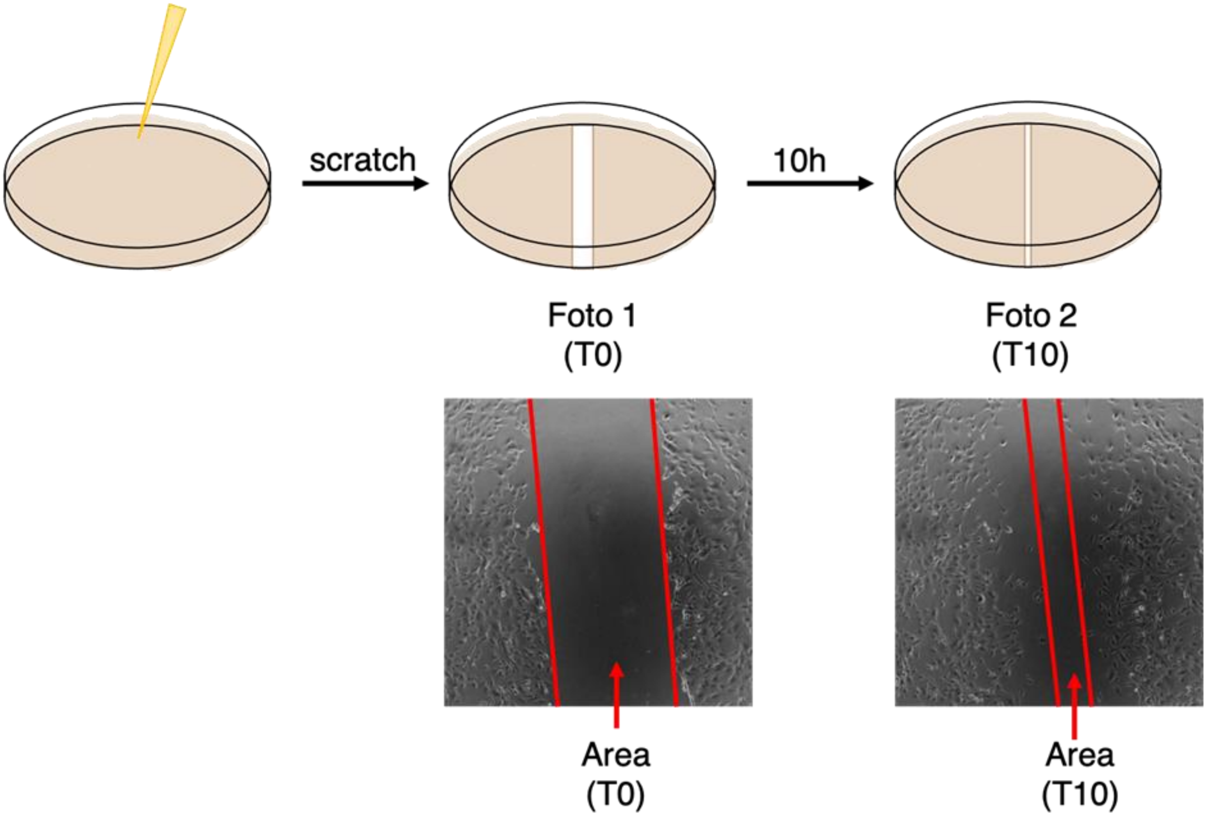

Figure S3. Schematic representation of experimental set-up for the scratch/wound-closure assay. Confluent monolayers were mechanically scratched with a yellow pipette tip (10 µl–200 µl) before treatment was applied. Images of each scratch were taken right after the scratch was made (T0) and 10h thereafter (T10) with an automated Olympus IX81 microscope (Olympus, Volketswil, CH). Area of wound closure was determined by using the software ImageJ, and relative wound closure was calculated as follows: Area(T0)-Area(T10)) / Area(T0). Representative images are depicted. The schematic figure is in part reproduced from our previously published work (Kurmann, L.; Okoniewski, M.; Dubey, R.K. Estradiol Inhibits Human Brain Vascular Pericyte Migration Activity: A Functional and Transcriptomic Analysis. *Cells* **2021**, *10*, 2314. https://doi.org/10.3390/cells10092314).
